# A novel surgical technique for bleeding duodenal varices after failure of balloon-occluded retrograde transvenous obliteration: a case report

**DOI:** 10.1186/s40792-016-0192-z

**Published:** 2016-06-27

**Authors:** Go Anegawa, Kenji Sumi, Atsushi Miyoshi, Kenji Kitahara, Seiji Satou

**Affiliations:** Department of Surgery, Saga-Ken Medical Centre Koseikan, 400 Kase-machi Nakabaru, Saga, 840-8571 Japan

**Keywords:** Duodenal varices, Balloon-occluded retrograde transvenous obliteration, Endoscopic band ligation, Embolization, Cirrhosis

## Abstract

**Background:**

Duodenal varices are a low-frequency cause of gastrointestinal bleeding; however, greater than 40 % mortality has been reported after the initial bleeding episode.

**Case presentation:**

This report describes a 72-year-old woman with bleeding duodenal varices treated by surgery after failure of balloon-occluded retrograde transvenous obliteration (B-RTO). The patient presented with profuse melena. Emergent upper endoscopy was immediately performed, and bleeding duodenal varices in the second portion of the duodenum were seen. Endoscopic band ligation was attempted first followed by B-RTO; however, the combined procedures failed. Laparotomy under general anesthesia was then performed, and the venous collaterals were cannulated using an 18-gauge needle. Following intraoperative angiography, the venous collateral was ligated on the peripheral side of the needle entry point, and ethanolamine oleate was injected into the afferent collateral vessel. Endoscopic examination on postoperative day 4 showed embolization of the duodenal varices. The patient was discharged on postoperative day 11.

**Conclusions:**

This technique is simple and effective, and we believe it is a potential alternative surgical treatment for duodenal varices with portal hypertension.

## Background

Duodenal varices are a low-frequency cause of gastrointestinal bleeding; however, greater than 40 % mortality has been reported after the initial bleeding episode [1, 2]. Several treatments exist to control bleeding including interventional radiology, endoscopic therapy, and surgical modalities (e.g., variceal ligation, duodenal resection, and extrahepatic portosystemic shunts) [3]. Endoscopic therapy is often the first choice for bleeding duodenal varices and can include endoscopic injection sclerotherapy and the use of *N*-butyl-2-cyanoacrylate [4, 5]. However, these procedures are specialized and performed in few institutions in Japan. Endoscopic band ligation is easy and useful for temporary hemostasis; however, the frequency of variceal re-bleeding is high and additional therapy including interventional radiology and/or surgical treatment is necessary [5, 6]. We present a novel surgical technique for bleeding duodenal varices after failure of balloon-occluded retrograde transvenous obliteration (B-RTO).

## Case presentation

**Fig. 1:**
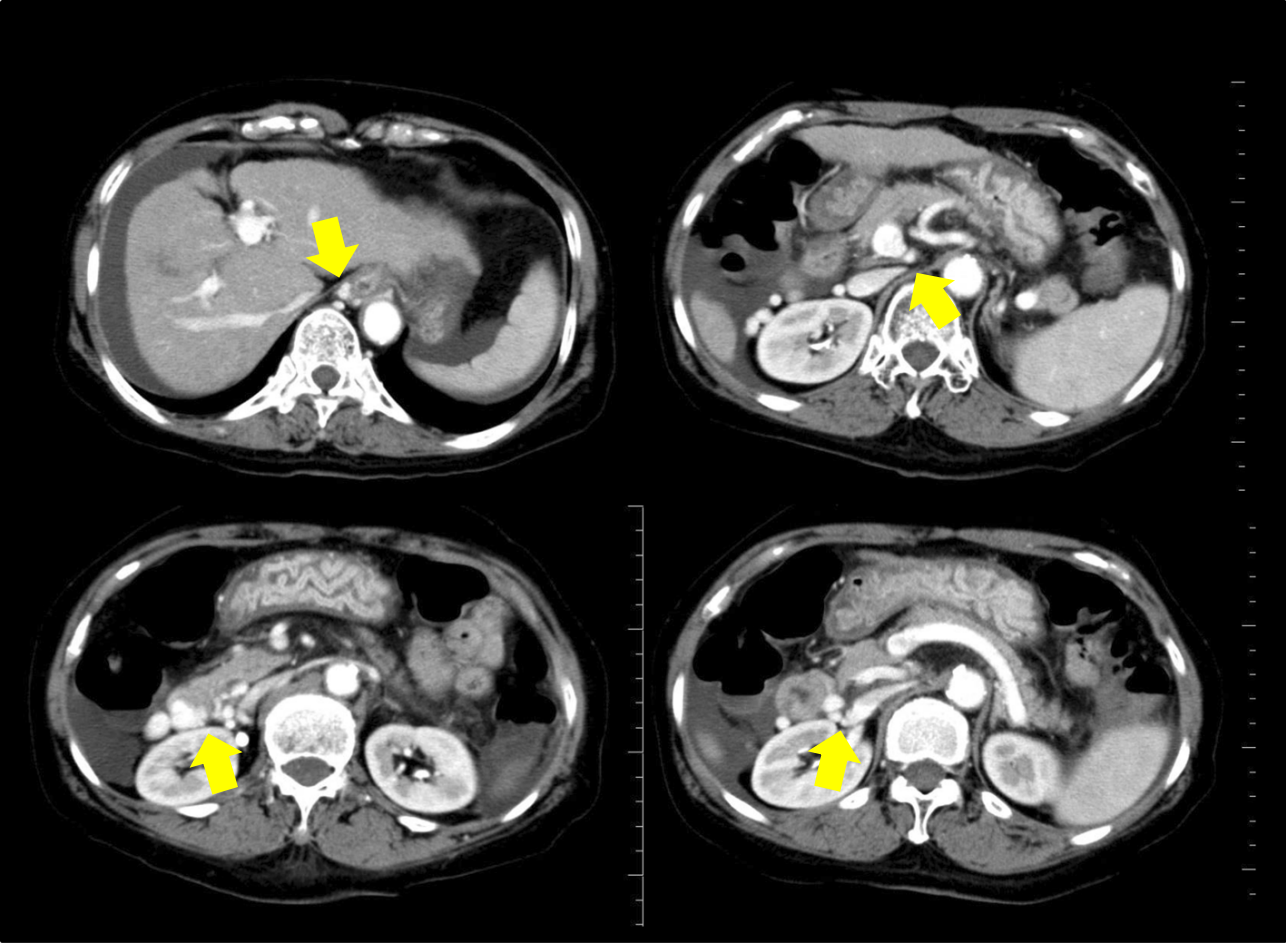
Initial abdominal computed tomographic images showing paraesophageal varices and venous collaterals around the duodenum. Extravasated contrast is seen in the second portion of the duodenum. The afferent collateral vessel originated directly from the main portal vein, and the efferent collateral vessel drained into the inferior vena cava via the ovarian veins

**Fig. 2:**
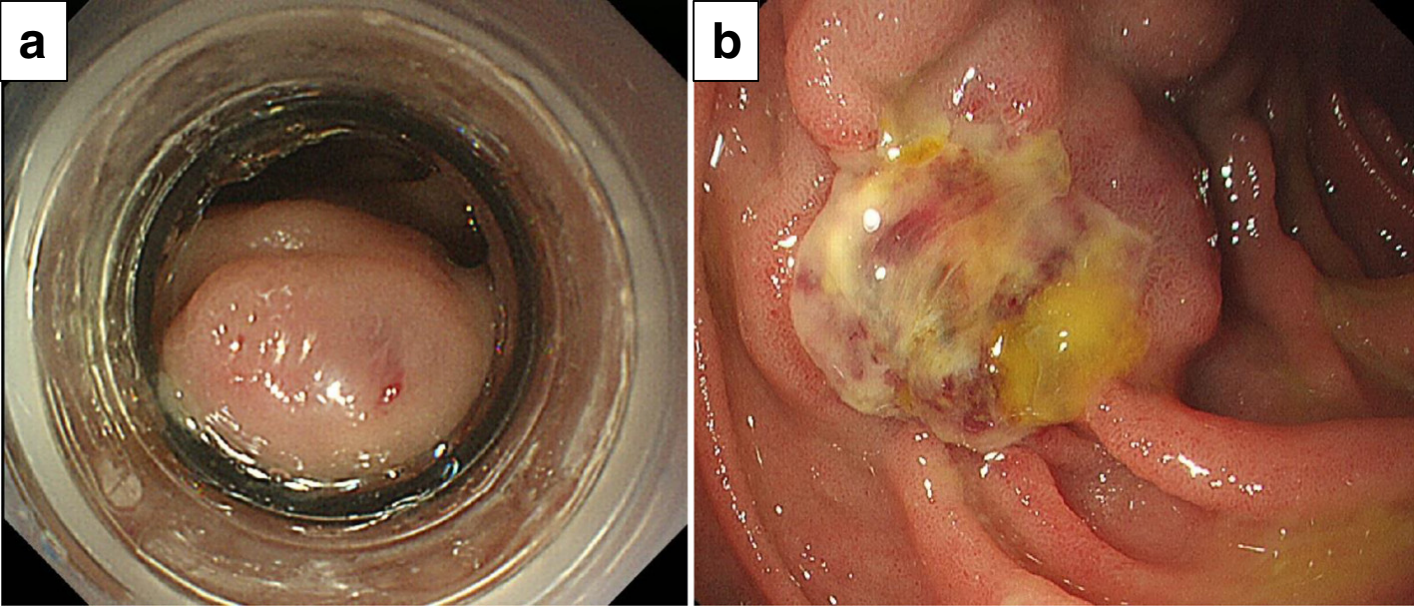
**a** Endoscopy image showing duodenal varices in the second portion of the duodenum. **b** Photograph showing the endoscopic findings on postoperative day 4 confirming embolization of the duodenal varices

**Fig. 3:**
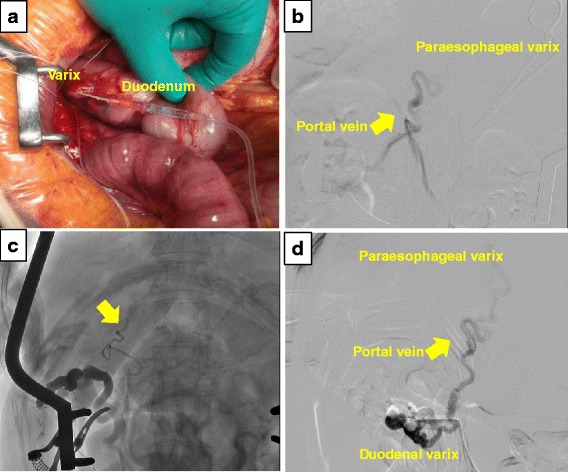
**a** Photograph showing cannulation of the venous collaterals surrounding the duodenum using an 18-gauge needle. **b** Angiogram showing the portal vein (*arrow*) and paraesophageal varix. **c** Angiogram with clump of central side of the needle point showing the efferent collateral vessel draining into the inferior vena cava via the ovarian veins (*arrow*). **d** Angiogram with clump of peripheral side of the needle point showing the duodenal varices, portal vein, and paraesophageal varices

A 72-year-old woman with liver cirrhosis confirmed based on hepatitis C virus serology presented with profuse melena. Initial abdominal computed tomography showed paraesophageal varices and venous collaterals around the duodenum. Extravasated contrast was identified in the second portion of the duodenum with the afferent collateral vessel originating directly from the main portal vein. The efferent collateral vessel drained into the inferior vena cava via the ovarian veins (Fig. 1). Emergent upper endoscopy was immediately performed, and bleeding duodenal varices in the second portion of duodenum were identified (Fig. 2a). Endoscopic band ligation was attempted first, and B-RTO was prepared simultaneously. Unfortunately, B-RTO following EBL failed because of unsuccessful cannulation of the ovarian vein, and there was a surgical treatment request. Laparotomy was performed under general anesthesia, and the venous collaterals around the second portion of the duodenum were identified and cannulated using an 18-gauge needle (Fig. 3a). Following cannulation of the venous collaterals, intraoperative angiography revealed portal vein and paraesophageal varices (Fig. 3b). The efferent venous collateral was ligated to the side of the needle entry point, and intraoperative angiography revealed duodenal varices, portal vein, and paraesophageal varices (Fig. 3d). The amount of contrast injected into the portal vein during intraoperative angiography was estimated. The afferent venous collateral was then ligated centrally over the needle entry point, and intraoperative angiography identified the ovarian veins and inferior vena cava (Fig. 3c). The remaining venous collateral was then ligated to the side of the needle entry point, and 6 mL ethanolamine oleate (EO; Grelan, Tokyo, Japan) solution with iopamidol 300 (5 % EOI; Schering, Berlin, Germany), which was the amount of contrast injected into the portal vein during the previous intraoperative angiography, was slowly injected into the afferent collateral vessel under intraoperative angiography, completing the operation. Endoscopic examination on postoperative day 4 showed embolization of the duodenal varices (Fig. 2b). The patient was discharged on postoperative day 11; however, she died because of liver failure 3 months after this operation without recurrence of the melena.

## Conclusions

From our experience, we believe that direct injection of ethanolamine oleate into the afferent collateral vessel under laparotomy could be an alternative surgical hematemesis for rupture of duodenal varicose vein when EVL and B-RTO were failed.

## Abbreviations

B-RTO, balloon-occluded retrograde transvenous obliteration

## References

[1] D’Imperio N, Piemontese A, Baroncini D, Billi P, Borioni D, Dal Monte PP (1996). Evaluation of undiluted N-butyl-2-cyanoacrylate in the endoscopic treatment of upper gastrointestinal tract varices. Endoscopy.

[2] Khouqeer F, Morrow C, Jordan P (1987). Duodenal varices as a cause of massive upper gastrointestinal bleeding. Surgery.

[3] Kakizaki S, Toyoda M, Ichikawa T, Sato K, Takagi H, Arai H (2010). Clinical characteristics and treatment for patients presenting with bleeding duodenal varices. Dig Endosc.

[4] Barbish AW, Ehrinpreis MN (1993). Successful endoscopic injection sclerotherapy of a bleeding duodenal varix. Am J Gastroenterol.

[5] Ota K, Shirai Z, Masuzaki T, Tanaka K, Higashihara H, Okazaki M (1998). Endoscopic injection sclerotherapy with N-butyl-2-cyanoacrylate for ruptured duodenal varices. J Gastroenterol.

[6] Ohta M, Yasumori K, Saku M, Saitsu H, Muranaka T, Yoshida K (1999). Successful treatment of bleeding duodenal varices by balloon-occluded retrograde transvenous obliteration: a transjugular venous approach. Surgery.

